# Immune infiltrates impact on the prediction of prognosis and response to immunotherapy of melanoma patients

**DOI:** 10.1186/1479-5876-13-S1-P12

**Published:** 2015-01-15

**Authors:** Angela Vasaturo, Dagmar Verweij, Lucie Heinzerling, Jolanda de Vries, Willeke Blokx, Carl G Figdor

**Affiliations:** 1 Department of Tumor Immunology, Radboud Institute for Molecular Life Sciences, Nijmegen, Netherlands; 2 Department of Dermatology, University Hospital of Erlangen, erlangen, Germany; 3 Department of Pathology, Radboud University Medical Center, Nijmegen, The Netherlands

## Background

Melanoma is a highly malignant melanocyte-derived tumor and its incidence is increasing at outstanding rate. Despite specific therapies have been explored for many years, no effective therapeutic options have been developed. Vaccination strategies, including Dendritic Cells (DC) based immunotherapy, are consistently increasing the proportion of cancer patients with anti-vaccine immune responses although the number of patients with increased overall survival is still limited. The efficacy of the immunotherapy is mainly dependent on tumor microenvironment –immune system interactions.

Our aim is to evaluate the host immune response to melanoma by quantifying the density and location of T cells in primary tumors of patients treated with DC immunotherapy and correlate them with clinical variables such as overall survival (OS).

## Materials and methods

We collected 60 FFPE primary tumors from melanoma patients treated with DC immunotherapy. Serial sections (4 micron in thickness) were stained with CD3, CD8 and CD45RO antibodies. Haematoxylin was used as a counterstain and Nova Red for the immunohistochemical stain. All the slides were digitalized and an automated quantitative analysis was performed in order to evaluate the density and location of two lymphocyte populations, cytotoxic (CD8) and memory (CD45RO) T cells. Of all samples the clinical outcome of the patient is known.

## Results

The immunohistochemical analysis of primary melanoma using a small set of patients resulted in significant differences between short (OS<12 months) and long survivors (OS>24 months). A high degree of T cells infiltration was seen in the tumor area of both patients, responding and non-responding to DC immunotherapy. However, the location but not the density of TILs was significantly different in the two cohorts of patients and showed a strong correlation with clinical response to DC vaccination.

## Conclusions

Immune cells within melanoma tumors may have a prognostic value and clinical significance as a predictor of patient outcome and response to DC immunotherapy in melanoma patients.

